# Serum vitamin D level in COVID-19 patients and its correlation with disease severity

**DOI:** 10.1186/s43166-022-00155-9

**Published:** 2022-10-07

**Authors:** Caroline S. Morad, Reem A. Habeeb, Esraa T. Yassin, Salma A. Khalil

**Affiliations:** Internal Medicine Department, Rheumatology Division, Ain Shams University, Cairo, Egypt

**Keywords:** COVID-19, Serum vitamin D, C-reactive protein, Serum ferritin, Severity, Oxygen demand

## Abstract

**Background:**

Severe coronavirus disease 2019 (COVID-19) infections are associated with increased levels of C-reactive protein (CRP) and several pro-inflammatory cytokines leading to cytokine storm. Vitamin D has been proved to be associated with biological activities of the innate and adaptive immune systems. There is a growing number of data showing an association between serum vitamin D level and the different clinical outcomes of COVID-19 infection. Our aim is to evaluate the relation between serum vitamin D levels and the severity and mortality of COVID-19 infection in an Egyptian cohort.

**Results:**

The study included 80 COVID-19 patients, 38 males (47.5%) and 42 females (52.5%), with a mean age of 52 ± 11.4 years (18–80 years). The serum vitamin D levels ranged between 2 and 30 ng/mL with a mean of 12.05 ± 9.04. Patients who were intubated had the lowest levels of serum vitamin D (7.26 ng/ml ± 5.21), while patients who had no need for oxygen supply had the highest levels (20.00 ng/ml ± 9.23) (*P* = 0.025). A highly significant negative correlation was found between serum vitamin D level and each of CRP and serum ferritin (*r* = − 0.346 and − 0.313) (*P* = 0.002 and 0.005). Of the enrolled 80 patients, 63 (79%) recovered (group 1) and 17 (21%) died (group 2). Group 2 had significantly lower vitamin D levels (6.17 ng/mL ± 3.22) and hemoglobin (10.75 ± 1.74) than group 1 patients (13.63ng/mL ± 9.46) (12.10 ± 1.85) (*P* = 0.002 and 0.009 respectively).

**Conclusion:**

Serum vitamin D levels are significantly lower in patients needing mechanical ventilation, and in deceased patients, and are inversely related to the inflammatory markers CRP and serum ferritin, suggesting a relation between vitamin D insufficiency and poor COVID-19 outcome.

## Background

The coronavirus disease 2019 (COVID-19) caused by severe acute respiratory syndrome coronavirus 2 (SARS-CoV-2) was first reported in the city of Wuhan (China) in December 2019 and was declared a global pandemic by the World Health Organization (WHO) on March 11, 2020 [1].

Over the following months, COVID-19 has placed a huge burden on healthcare systems worldwide, posing serious threats to global health. Older age and underlying comorbid conditions have emerged as major risk factors for mortality related to COVID-19 [2]. Patients with severe manifestations of COVID-19 exhibit significantly increased circulating levels of C-reactive protein and several pro-inflammatory cytokines and chemokines that all lead to cytokine storm and eventually death [3].

Over the last decade, vitamin D has been proved to be associated with the biological activities of the innate and adaptive immune systems, as well as induction of the anti-inflammatory pathways in response to different pathogens [4]. Vitamin D deficiency represents a global pandemic, affecting individuals across all age groups worldwide. There is a growing number of data showing an association between serum 25-hydroxyvitamin D level and the different clinical outcomes of COVID-19 infection, particularly the related severity and mortality [5].

The aim of this study was to evaluate the relation between serum vitamin D and severity and mortality of COVID-19 infection in an Egyptian cohort.

## Methods

Eighty COVID-19 patients were randomly recruited from the wards and the intensive care units (ICU) of the Ain Shams University Hospitals including the El Medany Hospital, El Demerdash Hospital, and El Obour Specialized Hospital. Approval was taken from the Ethical Committee of Scientific Research, Ain Shams University. After giving their informed written consent, patients were enrolled in this cross-sectional observational study during the period between June 2021 and December 2021.

All recruited patients were confirmed COVID-19 cases who had a positive result on real-time reverse transcriptase-polymerase chain reaction (RT-PCR) of nasopharyngeal and oropharyngeal swab specimens. Patients with malignancy, chronic kidney disease, chronic liver disease, parathyroid disease, current use of vitamin D or calcium supplements, pregnancy, and obesity were excluded.

All patients were subjected to detailed medical history with emphasis on symptoms of COVID-19 besides associated medical comorbidities (hypertension, diabetes, ischemic heart diseases, and chronic obstructive pulmonary diseases (COPD)). Clinical assessment of each participant was done as well as recording the need and type of oxygen supply and the fate of the disease either recovery or mortality. To study the relation between vitamin D levels and mortality, patients were divided into 2 groups. Group 1 included patients who recovered from COVID-19 infection, and group 2 included the deceased patients. Comparison was done between both groups as regards laboratory data and serum vitamin D level.

Laboratory tests included complete blood count (CBC), C-reactive protein (CRP), serum ferritin, D-dimer, serum calcium, serum phosphorus, renal function tests (blood urea nitrogen (BUN) and creatinine), liver enzymes (alanine aminotransferase (ALT), and aspartate aminotransferase (AST)). Serum 25-hydroxycholecalciferol level was measured for all the participants on hospital admission. Blood samples were collected in anticoagulant-free tubes. Immediate centrifugation (3500 rpm) for 20 min at +4C and serum was stored at – 20 °C until analysis. Serum vitamin D levels were measured by automated competitive electrochemiluminescence protein binding assay method with an autoanalyzer (Beckman Coulter in DXI 800).

All patients underwent non-contrast-enhanced chest computed tomography (CT) that assessed the COVID-19 Reporting and Data System (CO-RADS) grading score. CO-RADS score grades the level of suspicion of pulmonary involvement of COVID-19 infection as follows:

CO-RADS 1: COVID-19 is highly unlikely, CT is normal, or there are findings indicating a non-infectious disease. CO-RADS 2: the level of suspicion of COVID-19 infection is low, and CT findings are consistent with other infections. CO-RADS 3: COVID-19 infection is unsure or indeterminate, and CT abnormalities indicate infection but are unsure whether COVID-19 is involved. CO-RADS 4: the level of suspicion is high, and most CT findings are suspicious but not extremely typical as unilateral ground glass, confluent, or multifocal consolidations without a typical location or any other typical findings. CO-RADS 5: the level of suspicion is high with typical CT findings [6].

### Statistical analysis

Data were entered to the Statistical Package for Social Science (SPSS) version 20. Qualitative data were presented as number and percentages, while quantitative data were presented as mean, standard deviations, and ranges. The comparison between 2 groups with qualitative data were done using chi-square test, and Fisher exact test was used when the expected count in any cell was < 5. The comparison between 2 quantitative data with parametric distribution was done using independent *T*-test. *P*-value ≤ 0.05 was considered significant.

## Results

The current study included 80 COVID-19 patients, 38 males (47.5%) and 42 females (52.5%), with a mean age of 52 ± 11.4 (18–80 years). Participants had different pre-existing comorbidities; 40 patients (50%) were diabetic, 41 patients (51.25%) were hypertensive, 16 patients (20%) had ischemic heart disease, and 13 patients (16.25%) had COPD. Chest CT revealed a CO-RADS 3 stage in 34 patients (42.5%), 25 patients (31.25%) had CO-RADS 4, and 21 patients (26.25%) had CO-RADS 5.

Of the enrolled patients, 23.75% were intubated, 30% were on nasal oxygen, 35% needed different modes of non-invasive oxygen therapy, and 11% did not need any oxygen therapy. Laboratory data of the included patients are shown in Table 1.

**Table 1:** Laboratory data of the COVID-19 patients

Laboratory data mean ± SD	COVID-19 patients All (*n* = 80)	Group 1 (recovered) (*n* = 63)	Group 2 (deceased) (*n* = 17)	*P*
Leucocytic count (×10^3^/μL)	9.366 ± 4.972	8.610 ± 3.647	12.171 ± 7.741	0.008*
Hemoglobin (g/dL)	11.816 ± 1.904	12.101 ± 1.857	10.759 ± 1.742	0.009*
Platelet count (×10^3^/μL)	241.038 ± 106.597	242.222 ± 110.796	236.647 ± 92.250	0.850
C-reactive protein (mg/L)	75.430 ± 69.311	73.405 ± 68.151	82.935 ± 75.141	0.618
Aspartate aminotransferase (IU/L)	52.538 ± 154.420	53.111 ± 172.554	50.412 ± 48.513	0.949
Alanine aminotransferase (IU/L)	37.113 ± 75.016	39.746 ± 84.223	27.353 ± 13.029	0.549
Blood urea nitrogen (mg/dL)	27.900 ± 19.467	24.476 ± 12.980	40.588 ± 31.641	0.002*
Creatinine (mg/dL)	0.880 ± 0.198	0.893 ± 0.198	0.829 ± 0.193	0.242
Sodium (mEq/L)	136.43 ± 83.974	136.127 ± 3.916	137.588 ± 4.094	0.180
Potassium (mEq/L)	4.145 ± 0.551	4.224 ± 0.550	3.853 ± 0.461	0.013*
Calcium (mg/dL)	8.638 ± 0.464	8.643 ± 0.423	8.621 ± 0.609	0.866
Phosphorous (mg/dL)	2.719 ± 0.650	2.686 ± 0.620	2.841 ± 0.761	0.385
Alkaline phosphatase (U/L)	77.175 ± 29.120	73.540 ± 28.887	90.647 ± 26.620	0.031*
D-dimer (mg/L)	1.321 ± 1.467	1.215 ± 1.276	1.712 ± 2.028	0.217
Serum ferritin (ng/mL)	1187.66 ± 1515.36	1159.02 ± 1667.54	1293.82 ± 740.08	0.747
Vitamin D (ng/mL)	12.050 ± 9.047	13.635 ± 9.465	6.176 ± 3.226	0.002*

Bold values are significant at *P* ≤ 0.05

The serum vitamin D levels of the enrolled COVID-19 patients ranged between 2 and 30 ng/mL with a mean of 12.05 ± 9.04. Serum vitamin D levels did not differ significantly in males and females (*P* = 0.58) nor in patients with different comorbidities. Although patients with different grades of CO-RADS staging on chest CT did not have significantly different levels of serum vitamin D level (*P* = 0.28), patients with CORAD 5 had the lowest vitamin D levels.

A significant difference of serum vitamin D level was found in patients on different modes of oxygen supply. Patients who were intubated (critical COVID-19 patients) had the lowest levels of serum vitamin D (7.26 ng/ml ± 5.21), while patients who had no need for oxygen supply (non-severe COVID-19) had the highest levels (20.00 ng/ml ± 9.23) (*P* = 0.025) (Table 2).

**Table 2:** Comparison between vitamin D levels in COVID-19 patients needing different modes of oxygen therapy

Oxygen therapy	Serum vitamin D level		ANOVA	
	*n*	Mean ± SD	*F*	*P*
No	9	20.000 ± 9.233	2.582	0.025*
Nasal	24	12.583 ± 9.578		
Face mask	15	14.133 ± 10.260		
Venturi mask	3	11.667 ± 0.577		
Continuous positive airway pressure	3	9.667 ± 5.859		
Non-rebreathing mask	7	9.714 ± 9.214		
Intubated	19	7.263 ± 5.216		

Bold values are significant at *P* ≤ 0.05

**Fig. 1:**
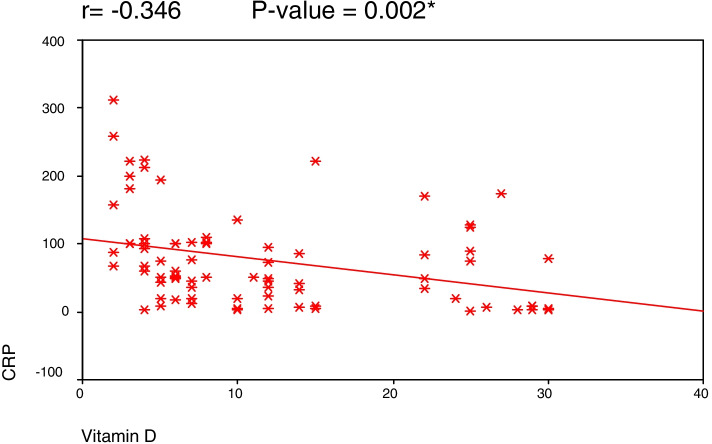
Correlation between C-reactive protein (CRP) and vitamin D level

**Fig. 2:**
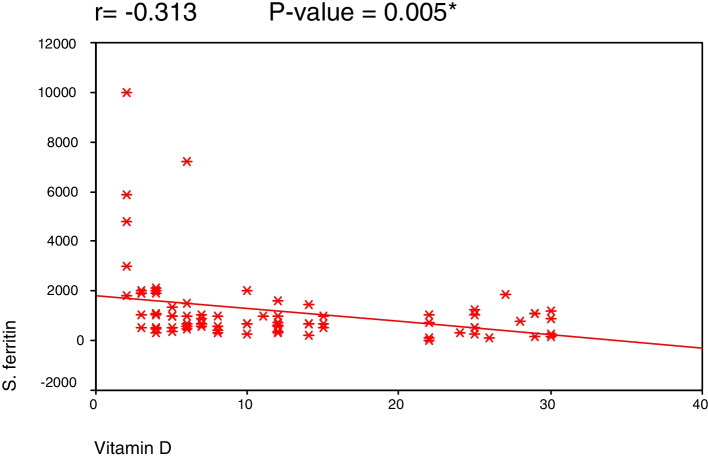
Correlation between serum ferritin and vitamin D level

A highly significant negative correlation was found between serum vitamin D level and each of CRP (Fig. 1), serum ferritin (Fig. 2), and alkaline phosphatase (*r* = − 0.346, − 0.313, and − 0.343, *P* = 0.002, 0.005, and 0.002 respectively).

Patients were divided into two groups according to the fate of COVID-19 illness. Of the enrolled 80 patients, 63 (79%) recovered (group 1) and 17 (21%) died (group 2). Gender did not affect the survival; 53% of the deceased patients were males, while 47% were females. The mean age of group 1 patients (50 ± 11 years) was not significantly lower than that of group 2 patients (52 ± 11 years). Mortality was not significantly affected by different comorbidities in the enrolled patients as shown in Table 3.

**Table 3:** Relation between comorbidities and mortality in the COVID-19 patients

		Mortality		Chi-square	
		Yes (*n* = 17)	No (*n* = 63)		
		*n* (%)	*n* (%)	*χ*^2^	*P*
Diabetes mellitus	Yes	8 (47.06)	32 (50.79)	0.075	0.785
	No	9 (52.94)	31(49.21)		
Cerebrovascular stroke	Yes	1 (5.88)	3 (4.76)	0.035	0.851
	No	16 (94.12)	60 (95.24)		
Ischemic heart disease	Yes	3 (17.65)	13 (20.63)	0.075	0.785
	No	14 (82.35)	50 (79.37)		
Hypertension	Yes	9 (52.94)	32 (50.79)	0.025	0.875
	No	8 (47.06)	31(49.21)		
Chronic obstructive pulmonary disease	Yes	3 (17.65)	10 (15.87)	0.031	0.860
	No	14 (82.35)	53 (84.13)		

*P*-value ≤ 0.05 is considered significant

Group 2 patients who did not survive had significantly lower vitamin D levels (6.17ng/mL ± 3.22) and hemoglobin (10.75 ± 1.74) than group 1 patients (13.63 ng/mL ± 9.46) (12.10 ± 1.85) (*P* = 0.002 and 0.009 respectively). In addition, group 2 patients had significantly higher levels of white blood cell count (WBCs), BUN, and alkaline phosphatase than group 1 patients (*P* = 0.008, 0.002, and 0.031 respectively) (Table 1).

## Discussion

During the COVID-19 pandemic period, researchers focus on deep survey of the modifiable factors which can reduce the severity of the COVID-19 infection. Vitamin D is known to play a key role in modulation of the immune response in infectious diseases. Different studies showed that severity of COVID-19 infection is directly related to vitamin D level [5, 7–13].

The current study included 80 COVID-19 hospitalized patients and investigated the relationship between serum vitamin D levels and the severity of COVID 19 disease. The mean age of the patients was 52 ± 11.4 years. This was not surprising and goes along with the work of Basaran et al. [7] (57.55 ± 17.89 years), as older COVID-19 patients are more likely to need hospitalization.

We found no significant difference in vitamin D levels according to age or sex. This finding goes along with that of Yosef et al. [14] who also conducted their study on an Egyptian cohort. In contrast, many other studies showed lower levels of vitamin D in older patients than younger patients and in females than males [15, 16].

In the present study, a significant difference in serum vitamin D level was found in patients on different modes of oxygen therapy. Patients who were intubated had the lowest levels of serum vitamin D level (7.26 ng/ml ± 5.21), while patients who had no need for oxygen supply had the highest levels (20.00 ng/ml ± 9.23) (*P* = 0.025). This goes along with the analyzed data of 144 patients in USA that proved that sufficient vitamin D level was independently significantly associated with lower need for invasive mechanical ventilation (odds ratio 0.96; *P* = 0.01) [10], while another study conducted in UAE reported that vitamin D levels were not correlated to the need for mechanical ventilation [8].

In this study, a decrease in vitamin D levels was associated with more severe COVID-19 cases, as evidenced by significantly higher blood levels of inflammatory markers CRP and serum ferritin (*P* = 0.005 and 0.002 respectively), suggesting that vitamin D might have a beneficial role on the systemic inflammatory state of this viral disease. Consistent with our data, in a cohort of 235 COVID-19 patients, Maghbooli et al. [17] showed that CRP levels were lower in patients with vitamin D > 30 ng/ml. Similarly, Saporano et al. [9] found a significant inverse correlation between vitamin D levels and each of CRP, D-Dimer, and interleukin 6 (IL6). In another study by Daneshkhah et al. [18], high levels of CRP were associated with hypovitaminosis D. On the other hand, Hafez et al. [8] found a significant inverse correlation between vitamin D level and both lactate dehydrogenase (LDH) and fibrinogen, but not CRP or ferritin.

Few studies also evaluated the relationship between D-dimer levels and serum vitamin D level. In contrast to our data that did not show a significant relation between vitamin D levels and D-dimer, the retrospective study by Demir et al. [19] on 227 COVID-19 patients showed that D-dimer was higher in the presence of low vitamin D levels. Similar results were reported by Giannini et al. [20] in a cohort of 91 patients.

Regarding the parameters affecting mortality, age, sex, and presence of different comorbidities did not differ significantly between survivors (group 1) and deceased patients (group 2). Similarly, Alsafar et al. [11] showed that gender and comorbidities did not contribute to the mortality. However, in a retrospective two-center cohort study in UK including 433 COVID-19 patients, non-survivors were significantly older and more likely to have comorbidities [21]. This goes in line with a Brazilian study that had indicated a statistically significant correlation between mortality and both diabetes mellitus and hypertension [12]. The inconsistency between different studies suggests the presence of several confounding factors affecting the results, so further well-controlled clinical trials are required.

Considering serum vitamin D levels, they were significantly lower in COVID-19 deceased patients. This comes in harmony with many studies. One study in Turkey revealed that a significantly higher ratio of the deceased patients had vitamin D deficiency compared to surviving patients (92.8% vs. 48.8%, *P* < 0.001) and proved vitamin D deficiency to be an independent predictor of mortality [13]. Another retrospective study conducted upon 185 COVID-19 patients in Germany found that vitamin D deficiency at the time of admission was associated with higher risk of mortality (*P* = 0.001) [22]. In a recent meta-analysis including six studies and 376 COVID-19-positive patients, poor prognosis defined as severe symptoms, ICU admission, or death was present in 150 patients who also showed significantly lower levels of vitamin D, compared with patients with good prognosis [23]. Notably, Campi et al. [24] found that a 1-ng/ml increase in vitamin D levels was associated with a decrease of risk of death of 4%. However, Chen et al. [25] reported that neither vitamin D deficiency (< 20 ng/ml) nor insufficiency (< 30 ng/ml) was associated with increased risk of COVID-19 in-hospital death. Another study conducted in Iran with a cohort of 329 cases of COVID-19 showed no relationship between vitamin D status and death rate [26]. Additionally, a cohort study using UK Biobank showed no evidence that the hazard of COVID-19 mortality was higher among participants with vitamin D insufficiency or deficiency [27].

Additionally, deceased patients had significantly higher levels of WBCS, and BUN (*P* = 0.008 and 0.002 respectively). This is compatible with the results of a retrospective single center study in China, concluding that WBC count at admission is significantly correlated with death in COVID-19 patients [28].

Moreover, group 2 patients had significantly lower hemoglobin and potassium levels than group 1 patients (*P* = 0.023 and 0.013 respectively). This is in agreement with a prospective study in Iran reporting that anemic patients were significantly more likely to develop poor outcomes of COVID-19, including ICU admission and death [29]. Exacerbation of inflammation by the cytokine storm which occurs during the SARS-CoV-2 infection can explain the higher degrees of anemia found in the deceased patients.

Our study has some limitations. Our sample size was relatively small. In addition, no dietary assessment was carried out, and therefore, information on dietary habits is lacking.

## Conclusion

In conclusion, serum vitamin D levels were significantly lower in patients needing mechanical ventilation and in deceased patients. Moreover, serum vitamin D levels were significantly inversely related to the inflammatory markers CRP and serum ferritin, advocating the favorable effect of vitamin D on morbidity and mortality. Our study has clinical implications, as it suggests that vitamin D may calm down the inflammatory cascades that lead to severe outcomes from COVID-19. Provided its low cost and availability; further investigation of vitamin D supplementation as a preventive and therapeutic strategy for COVID-19 with randomized trials is warranted.

## Data Availability

The datasets generated and analyzed during the current study are available from the corresponding author upon request.

## Abbreviations

ALT: Alanine aminotransferase
AST: Aspartate aminotransferase
BUN: Blood urea nitrogen
CBC: Complete blood count
CO-RADS: COVID-19 Reporting and Data System
COVID-19: Coronavirus disease
CRP: C-reactive protein
CT: Computed tomography
ICU: Intensive care unit
LDH: Lactate dehydrogenase
RT-PCR: Reverse transcriptase-polymerase-chain reaction
SARS-CoV-2: Severe acute respiratory syndrome coronavirus 2
SPSS: Statistical Package for Social Science
WBC: White blood cell count
WHO: World Health Organization
